# Towards potent but less toxic nanopharmaceuticals – lipoic acid bioconjugates of ultrasmall gold nanoparticles with an anticancer drug and addressing unit†

**DOI:** 10.1039/c8ra01107a

**Published:** 2018-04-19

**Authors:** Maciej Dzwonek, Dominika Załubiniak, Piotr Piątek, Grzegorz Cichowicz, Sylwia Męczynska-Wielgosz, Tomasz Stępkowski, Marcin Kruszewski, Agnieszka Więckowska, Renata Bilewicz

**Affiliations:** Faculty of Chemistry, University of Warsaw Pasteura 1 02-093 Warsaw Poland bilewicz@chem.uw.edu.pl +48 22 55 26 357; Czochralski Laboratory of Advanced Crystal Engineering, Biological and Chemical Research Centre, Faculty of Chemistry, University of Warsaw Żwirki i Wigury 101 02-089 Warsaw Poland; Center for Radiobiology and Biological Dosimetry, Institute of Nuclear Chemistry and Technology Dorodna 16 03-195 Warszawa Poland; Department of Molecular Biology and Translational Research, Institute of Rural Health Jaczewskiego 2 20-090 Lublin Poland

## Abstract

Modification of ultrasmall gold nanoparticles (AuNPs) with the lipoic acid derivative of folic acid was found to enhance their accumulation in the cancer cell, as compared to AuNPs without addressing units. The application of lipoic acid enabled the control of the gold nanoparticle functionalities leading to enhanced solubility and allowing for attachment of both the folic acid and the cytotoxic drug, doxorubicin. More robust attachment of doxorubicin to the nanoparticle through the amide bond resulted in toxicity comparable with that of the drug alone, opening a new perspective for designing more potent, but less toxic nanopharmaceuticals. The increased uptake was accompanied by pronounced nuclear accumulation and observable cytotoxicity. Doxorubicin binding *via* covalent amide bonds enhanced stability of the whole drug vehicle and provided much better control over doxorubicin release in the cell environment, as compared to physical adsorption or pH sensitive bonding commonly used for anthracycline carriers. Confocal microscopy revealed that the bond was stable in the cytoplasm for 22 h. The ability to slow down the rate of drug release may be crucial for the application in sustained anticancer drug delivery. Biological analyses performed using MTT assay and confocal microscopy confirmed that the ultrasmall AuNPs with the lipoic acid derivative of folic acid exhibit relatively low cytotoxicity, however when loaded with a chemotherapeutic, they cause a significant reduction in the cell viability.

## Abstract 2

Modification of ultrasmall gold nanoparticles (AuNPs) with the lipoic acid derivative of folic acid was found to enhance their accumulation in the cancer cell, as compared to AuNPs without addressing units.

## Graphical abstract

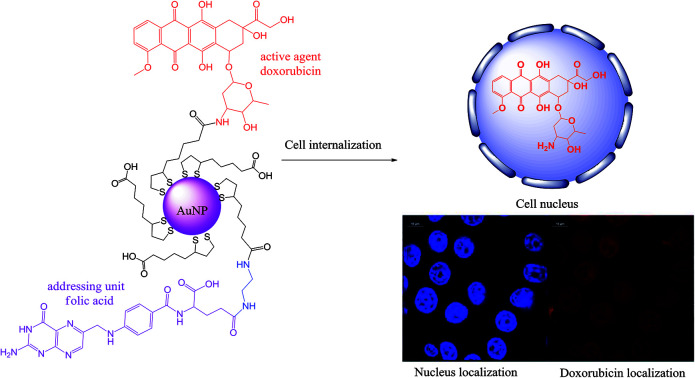

## Introduction

A modern targeted anticancer therapy utilizes antineoplastic drugs attached to biologically active molecules, which can selectively access the malformed tissue. A unique advantage of targeted therapy is the ability to destroy diseased tissues without damaging the healthy ones. Targeted delivery of cytotoxic drugs is particularly important for non-solid tumors or small, solid tumors at an early stage of development and small metastases. Targeted therapy requires finding an appropriate target on the tumor cells that can be targeted by a biologically active molecule that has an affinity for this target.

Gold nanoparticles possess some advantages such as a small size and ligand versatility due to the ease of surface functionalization and are therefore promising candidates for medical use.^1^ The acceptable toxicity of AuNPs enables their use for drug delivery to living organisms. The sizes of cells and organelles are in the micrometer range; therefore, nanoparticles are excellent candidates for transferring drugs into these biological structures.^2–4^ One-pot synthesis of gold nanoparticles gives the possibility of producing functional nanoparticles with a high monodispersity index and well characterized core and surface properties without additional steps of exchanging ligands,^5^ and appropriately chosen monolayers protecting the gold nanoparticles can provide water solubility and appropriate stability under physiological conditions.^6,7^

In the present study, folate was used as the AuNPs addressing unit. The folate receptors (FR) are overexpressed on the surface of many types of cancerous cells.^8^ The folate receptor binds folic acid (FA), as well as its derivatives, with a high affinity (*K*_d_ = 10^−10^ M)^9^ to maintain its steady-state level in fast growing cells. The drug and folic acid attached to the carrier are delivered directly to the cancerous cells and enter the cell *via* endocytosis. Folic acid consists of pteroic acid together with glutamic acid; however, the amino acid is not involved in the recognition process by the FR, which makes it suitable for modification.^10^

Solubility in aqueous solutions and a small size are crucial for a promising drug delivery vehicle. Usually, AuNPs that are modified with citrates (Turkevich-like method^11^) or covered with thiol derivatives of poly(ethylene)glycol (PEG) are used.^12,13^ The still limited practical usage of these AuNPs is connected with their relatively large diameters, leading to a very slow diffusion through the human tissue and thus a low efficiency in reaching the cancer cell environment. Another difficulty encountered is their slow removal from the organism. On the other hand, PEGylated nanocarriers as liposomes, micelles, solid lipid nanoparticles are found to induce the Accelerated Blood Clearance (ABC) phenomenon or have impact on the functioning of immune system.^14^

Among the various approaches to bind folic acid to gold nanoparticles, the most common is to prepare AuNPs and then to modify the ligands that are present at their surface using folic acid.^15,16^ The difficulty in binding nonmodified folic acid involves its low solubility in aqueous media. Moreover, the presence of 2 carboxylic groups in its structure does not facilitate conjugation following AuNPs formation, even though the gamma-COOH group is more active. The AuNPs structure may also be affected by the harsh changes of the conditions needed for deblocking, purifying and separation of the product.

Our approach differs from the common approach in that, first, the lipoic acid derivative of folic acid is synthesized, and then it is used directly during the synthesis of gold nanoparticles to form the AuNPs protective layer.^16^ Using a previously synthesized folic acid derivative that is conjugated with lipoic acid through the diamine linker removes all of the mentioned difficulties, allows better control of the whole procedure and increases the population of folic acid molecules present at the AuNPs surface. A good solubility of the AuNPs is achieved here by covering them with lipoic acid (LA) that is naturally present in both prokaryotic and eukaryotic cells.

Doxorubicin (Dox, trade names Adriamycin®, Rubex®, Scheme S1†) was covalently bound to the modified AuNPs. Dox is often used for the treatment of various types of cancers, *e.g.*, leukemia, Hodgkin's lymphoma, bladder, breast, stomach, lung, ovarian, thyroid, soft tissue sarcoma, and multiple myeloma.^17–19^ Dox is a potent drug; however, it possesses several disadvantages, including rapid excretion and numerous side effects, with the main and the most important being cardiac toxicity attributed to highly reactive oxygen species (ROS) production.^19,20^ Therefore, we propose a novel way of preparing targeted AuNPs carriers of Dox with a protective layer consisting of the lipoic acid (LA) and the lipoic acid derivative of the folic acid (LAFA); using microscopic imaging and cytotoxicity analysis, we demonstrate the successful uptake of the drug by cancer cells, its nuclear accumulation, and cellular apoptosis.

## Experimental section

### Materials and methods

#### Chemical and cell culture reagents

Tetrachloroauric acid trihydrate (HAuCl_4_·3H_2_O), tetraoctylammonium bromide (TOAB), lipoic acid (LA), folic acid (FA), sodium borohydride (NaBH_4_), *N*-hydroxysuccinimide (NHS), 1-ethyl-3-(3-dimethylaminopropyl)carbodiimide (EDC), ethylenediamine (NH_2_)_2_(CH_2_)_2_, *t*-butyloxycarbonyl anhydride (Boc_2_O), dimethylsulfoxide (DMSO), *N*,*N*′-dicyclohexylcarbodiimide (DCC), triethylamine (Et_3_N), *N*-disuccinimydyl carbonate, *N*,*N*-diisopropylethylamine (DIPEA), doxorubicin (Dox) were purchased from Sigma Aldrich and used without further purification.

Tetrahydrofuran (THF), dimethylformamide (DMF), dichloromethane (DCM), diethyl ether (Et_2_O), ethyl acetate (EtOAc), chloroform (CHCl_3_), sodium carbonate (Na_2_CO_3_), anhydrous magnesium sulfate (MgSO_4_), acetone and any other solvents were obtained from Avantor, Poland. All glassware was carefully cleaned with an acidic solution of KMnO_4_ (overnight), piranha solution (H_2_SO_4_ : H_2_O_2_ : H_2_O, 1 : 1 : 3) (2 h), rinsed with large amounts of Milli-Q water and dried.

EMEM media was a product of the American Type Tissue Culture Collection (ATCC, Rockville, MD). RPMI-1640 without folic acid was purchased from Thermo Fisher Scientific (USA). 3-(4,5-Dimethyl-2-thiazolyl)-2,5-diphenyl-2*H*-tetrazolium bromide (MTT) were obtained from Sigma Aldrich. Fetal calf serum was a product of Biological Industries (Israel).

### Instrumentation

Transmission electron microscopy (TEM) images were carried out using a FE-TEM Libra 120 Carl Zeiss microscope at a 120 kV acceleration voltage. The samples were placed onto a carbon coated copper microgrid (400 mesh) that was dried overnight. At least 5 different sample of nanoparticles were prepared, and from each grid at least 3 pictures were taken. The diameters of the cores of the nanoparticles were determined for at least 300 nanoparticles in each image.

The UV-Vis spectra were recorded using a Perkin Elmer Lambda 25 UV-Vis spectrophotometer.

Thermogravimetric measurements (TG) were carried out using a Netzsch STA 449 F1 Jupiter thermobalance. All samples were heated at a rate of 5°C min^−1^ up to 760 °C in an argon atmosphere. Argon (99.5%) was obtained from Air Products (Poland).

The electrochemical experiments were performed using a CHI 750D electrochemical workstation (CHI Instruments Inc., Austin, Texas) in the three-electrode arrangement employing a platinum foil counter electrode, an Ag/AgCl (KCl sat.) reference electrode and a glassy carbon disc electrode (BASI) as a working electrode.

All ^1^H and ^13^C NMR spectra were performed using a Bruker AVANCE 300 MHz spectrometer and the mass spectra were recorded with a Quattro LC mass spectrometer.

### Ligands synthesis – lipoic derivative of folic acid (LAFA)

Lipoic acid (LA) provides both a thiol function for covalent attachment to AuNPs and a carboxylic function that assures water solubility of LA functionalized nanoparticles. Folic acid (FA), as well as most of its derivatives, is poorly soluble in most organic solvents, which causes synthetic difficulties related mostly to product purification. FA contains two carboxylic groups that can undergo a nucleophilic substitution reaction. Therefore, the separation and purification of the main γ-substituted product from α-conjugates, bi-conjugates or unreacted FA are laborious.

**Scheme 1:**
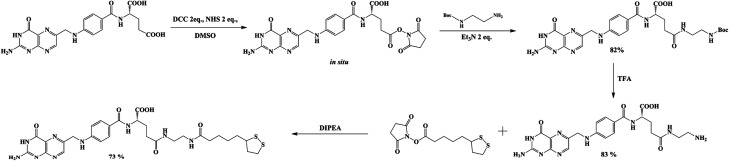
General scheme of lipoic–folic (LAFA) derivative synthesis.

**Scheme 2:**
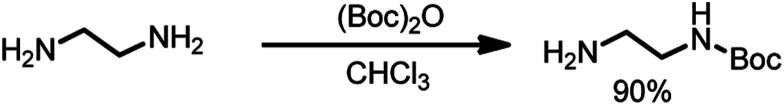
Protection of ethylenediamine using *t*-butyloxycarbonyl anhydride.

The preparation of LAFA (Scheme 1) starts from regioselective coupling of γ-carboxylic function of FA with *N*-Boc-ethylenediamine in conditions described by Trindade and co-workers.^21^ Next, Boc-group deprotection followed by the coupling of the resulting amine with *N*-hydroxysuccinimidyl liponate (LA-NHS) leads to a good yield of LAFA. The LA-NHS was obtained by the reaction of LA with *N*,*N*-disuccinimidyl carbonate (NHS) and *N*,*N*-diisopropylethylamine in deoxygenated acetone.^22^ The whole procedure of obtaining LAFA is depicted in Scheme 1, and each step is described in detail below. The respective NMR spectra are provided in ESI, S2† (Scheme 2).

In a round-bottomed flask 20 mL (0.3 mole, 10 eq.) of ethylenediamine in 200 mL of dry chloroform was dissolved and stirred at 0 °C. Then, a solution of Boc_2_O (6.5 g, 1 eq.) in 150 mL of chloroform was added dropwise. The reaction mixture was stirred for 24 hours at room temperature, and then the solvent was evaporated; the residue was re-dissolved in a solution of 2 M Na_2_CO_3_ (80 mL) and extracted four times with 60 mL of chloroform. The organic phases were combined, dried over MgSO_4_ and filtered, and the solvent was evaporated. *N*-Boc-Ethylenediamine (4.27 g, 90% yield) was isolated as a colorless oil (Scheme 2).

^1^H NMR (300 MHz, CDCl_3_) *δ*: 4.94 (s, 1H), 3.17–3.11 (q, 2H), 2.79–2.72 (t, 2H), 1.42 (s, 9H), 1.16 (s, 2H).

**Scheme 3:**
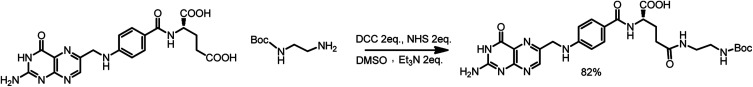
Modification of folic acid by protected ethylenediamine.

First, 640 mg of folic acid (1.45 mmole, 1 eq.) was dissolved in 25 mL of DMSO (30 minutes with mild heating). Then, 308 mg (2 eq.) of *N*-hydroxysuccinimide and 552 mg (2 eq.) of DCC were successively added. The reaction mixture was stirred for 16 h at room temperature, after which the urea precipitate was filtered through the pad of Celite. To the filtrate, 0.376 mL (2 eq.) of triethylamine and 429 mg (2 eq.) of *N*-Boc-ethylenediamine dissolved in 5 mL of DMSO were added. The mixture was stirred overnight, and then it was added to the mixture of 20% acetone in diethyl ether. The thin yellow precipitate was centrifuged, washed four times with acetone and four times with diethyl ether and was finally dried under vacuum (690 mg, 82% yield) (Scheme 3).

^1^H NMR (300 MHz, DMSO) *δ*: 11.46 (s, 1H), 8.64 (s, 1H), 8.20–7.86 (m, 2H), 7.68–7.62 (m, 2H), 7.30–6.72 (m, 3H), 6.66–6.63 (d, 2H), 4.48 (s, 2H), 4.27 (m, 1H), 3.09–2.85 (m, 4H), 2.28–1.85 (m, 4H), 1.35 (s, 9H).

^13^C NMR (300 MHz, DMSO) *δ*: 172.72, 166.68, 161.64, 156.08, 154.18, 151.23, 151.09, 148.57, 129.66, 129.32, 128.31, 121.59, 111.87, 78.27, 52.84, 46.43, 46.02, 40.94, 39.27, 32.88, 31.51, 28.68, 9.86.

MS (E^−^): 582.40.

**Scheme 4:**
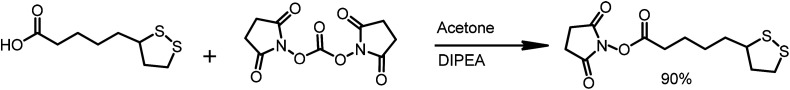
Activation of lipoic acid by *N*-disuccinimydylcarbonate.

The α-lipoic acid (2.0 g, 9.7 mmole) was dissolved in deoxygenated acetone (50 mL). The solution was protected from direct light by covering the reaction flask with aluminum foil. *N*,*N*-Disuccinimidyl carbonate (3.1 g, 1.25 eq.) and *N*,*N*-diisopropylethylamine (DIPEA, 2 mL, 1.25 eq.) were sequentially added and the reaction was stirred overnight at room temperature in an argon atmosphere. Then, solvent was evaporated, and the solid residue was re-dissolved in a mixture of water and DCM. The layers were separated, and the organic layer was washed with water (10 mL). The organic phase was dried over MgSO_4_ and was filtered. The solvent was removed *in vacuo*, and 2.65 g of LA-NHS in the form of a white solid was obtained (90% yield) (Scheme 4).

**Scheme 5:**
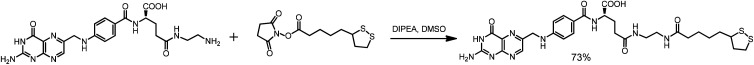
Conjugation of lipoic and folic acid derivatives.

^1^H NMR (300 MHz, CDCl_3_) *δ*: 3.62–3.53 (m, 1H), 3.19–3.09 (m, 2H), 2.83 (s, 4H), 2.66–2.60 (m, 2H), 2.5–2.43 (m, 1H), 1.96–1.89 (m, 1H), 1.81–1.54 (m, 6H) (Scheme 5).

Diisopropylethylamine (270 μL, 4 eq.) and *N*-hydroxysuccinimidyl liponate were added to a solution of ethylenediamine-folate (200 mg, 0.41 mmole) in 5 mL of DMSO and stirred until a clear solution was obtained (∼3 h). The solution was protected from direct light by covering the reaction flask with foil. The reaction mixture was poured into a mixture of 20% acetone in diethyl ether. The thin yellow precipitate was carefully centrifuged, washed six times with acetone and four times with diethyl ether and finally dried under vacuum (202 mg, 73% yield) (Scheme 5).

^1^H NMR (300 MHz, DMSO) *δ*: 8.63 (s, 1H), 8.14–7.88 (m, 2H), 7.68–7.62 (m, 2H), 7.17 (bs, 2H), 6,91 (bs, 2H), 6.65–6.63 (m, 2H), 4.48 (bs, 2H), 4.26 (bs, 1H), 3.59–3.49 (m, 1H), 3.41–3.34 (m, 1H) 3.36–2.92 (m, 6H), 2.89–2.82 (m, 2H), 2.26–1.80 (m, 8H), 1.69–1.39 (m, 6H), 1.36–1.23 (m, 3H).

^13^C NMR (300 MHz, DMSO) *δ*: 172.53, 167.10, 161.88, 160.00, 156.89, 154.66, 151.30, 148.96, 129.55, 129.19, 128.42, 121.83, 111.73, 65.38, 56.57, 46.39, 45.99, 35.71, 34.56, 28.79, 25.42, 19.20, 15.63, 9.97.

MS (ES^−^): 670.22.

### Gold nanoparticles synthesis

**Scheme 6:**
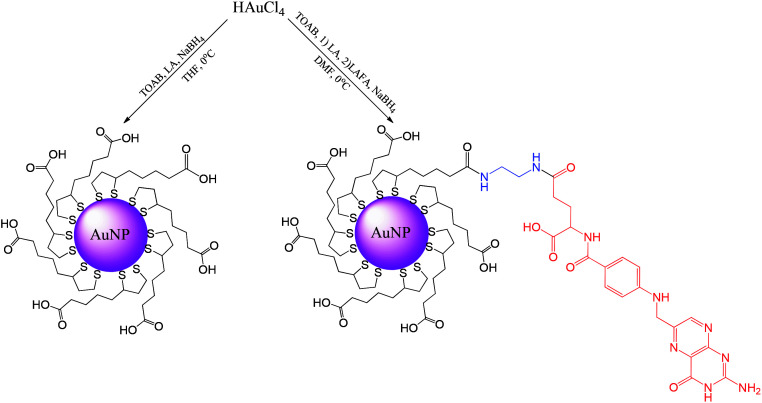
Synthesis of AuNPs modified with lipoic acid (AuNPs-LA) and lipoic acid/folic acid derivative (AuNPs-LA/LAFA).

Synthesis of ultrasmall gold nanoparticles (AuNPs-LA and AuNPs-LA/LAFA) was performed in tetrahydrofuran and dimethylformamide, respectively. During the formation of the polymeric structures, strict conditions as an argon atmosphere, low temperature and a dark environment were ensured. After the reduction of the gold precursor (HAuCl_4_ in the corresponding solvent) with sodium borohydride, the crude product was purified. A detailed description of the synthetic procedure, shown in Scheme 6, is provided below.

#### Synthesis of AuNPs-LA

First, 197 mg of HAuCl_4_·3H_2_O (0.5 mmole) was dissolved in 20 mL of anhydrous tetrahydrofuran, and then 328 mg of tetraoctylammonium bromide (TOAB, 0.6 mmole, 1.2 eq.) was added. Then, the reaction mixture was deaerated with argon and cooled in a water/ice bath for 30 min. Then, 413 mg of lipoic acid (LA, 2 mmole, 4 eq.) was dissolved in 15 mL of anhydrous THF and dropped into the reaction mixture and stirred for 12 hours in the dark and an argon atmosphere. Next, 189 mg (5 mmole, 10 eq.) of NaBH_4_ was dissolved in 10 mL of water and added carefully to the reaction mixture. The color of the mixture changed to dark brown, and the mixture was stirred for 3 hours. The dichloromethane/water mixture was added to facilitate phase separation. The organic phase was discarded, and the water phase was washed three times with dichloromethane, three times with ethyl acetate and two times with diethyl ether. The reaction mixture was preconcentrated under vacuum and was processed and concentrated using Vivaspin (MWCO 50 000 and MWCO 10 000) to remove any excess unbound lipoic acid, unreacted substrates and water-soluble products of NaBH_4_. The purification process was continued until the filtrate showed clear spectrum (only water) in UV-Vis. Then, the concentrate was filtered through a syringe filter (0.2 μm) and stored at 4 °C in the dark.

#### AuNPs-LA/LAFA

First, 98.5 mg of HAuCl_4_ (0.25 mmole) was dissolved in 20 mL of anhydrous dimethylformamide, and then 164 mg of tetraoctylammonium bromide (TOAB, 0.3 mmole, 1.2 eq.) was added. Then, the reaction mixture was degassed with argon and cooled in a water/ice bath for 1 hour. Then, 88.4 mg of folic derivative (LA-FA, ∼0.125 mmole, 0.5 eq.) was dissolved in 10 mL anhydrous DMF and was dropped into the reaction mixture and stirred for 18 hours in the dark and in an argon atmosphere. Then, the reaction mixture was again cooled in a water/ice bath, and 180 mg of lipoic acid (LA, 0.875 mmole, 3.5 eq.) was dissolved in 10 mL of DMF and dropped into the reaction mixture and stirred for 12 hours in the dark and in an argon atmosphere. Then, 94.5 mg of NaBH_4_ (2.5 mmole, 10 eq.) was dissolved in 5 mL of water, added carefully to the reaction mixture and stirred for 3 additional hours. Then, the dichloromethane/water mixture was added to facilitate the phase separation. When the phase was not separate, a few drops of 0.5 M NaOH was added. Then, the water phase was washed three times with dichloromethane, four time with ethyl acetate and three times with diethyl ether. Then, the reaction mixture was pre-concentrated under vacuum and the pH of the solution was adjusted to 7.0 with 0.1 M HCl. Then, the reaction was processed and concentrated with Vivaspin (MWCO 50 000 and MWCO 10 000) to remove any excess of the unreacted substrates. The purification process was continued until the filtrate showed a clear spectrum (only water) in UV-Vis. Then, the concentrate was filtered using a syringe filter (0.2 μm) and stored at 4 °C in the dark.

#### Conjugation of doxorubicin to AuNPs-LA/LAFA

**Scheme 7:**
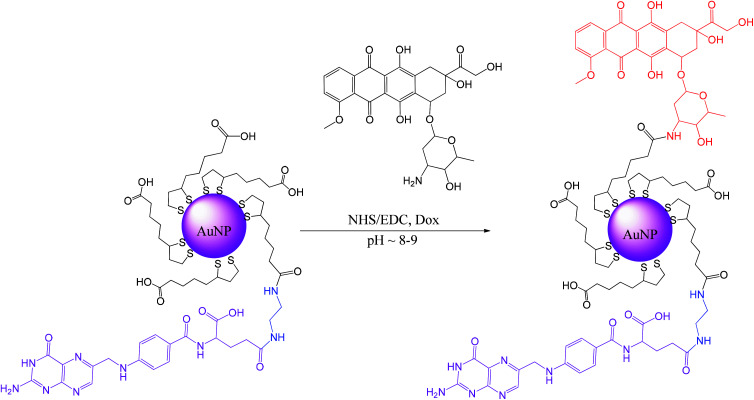
Conjugation of Dox with AuNPs-LA/LAFA.

Gold nanoparticles modified with lipoic acid and the folic acid derivative (AuNPs-LA/LAFA) were coupled with the drug doxorubicin. Scheme 7 presents the procedure of binding the drug, Dox, to the free LA-moieties of the AuNPs modified with LAFA.

First, 20 mL of AuNPs-LA/LAFA (∼0.4 mg mL^−1^) solution was adjusted to a pH of 8.0 with 0.01 M NaOH, and then the solution of coupling agents was added to 5 mL of water (15 μmole (1.7 mg) NHS/15 μmole (2.8 mg) EDC, 1.5 eq. according to Dox) and stirred for 2.5 h in the dark (aluminum foil). Then, the solution of doxorubicin (10 μmole (5.8 mg) Dox) was added to 5 mL of water. The mixture was stirred for another 5 hours and then was purified and concentrated using Vivaspin (10 000 MWCO). AuNPs-LA/LAFA–Dox were washed with water until the filtrate showed a clear spectrum (only water) in UV-Vis. Finally, the nanoparticles were collected and adjusted to a pH of 7.0 with 0.01 M HCl. Then, the product was filtered using a syringe filter (0.2 μm) and stored at 4 °C in the dark.

### Cell culture

Human cervical carcinoma KB cells (CCL-17), a derivative of HeLa cells, were purchased from the American Type Tissue Culture Collection (ATCC, Rockville, MD) and maintained according to the ATCC protocol in EMEM medium. When required, the EMEM medium was exchanged for a RPMI medium without folic acid to unblock the folic acid receptors. All media were supplemented with 10% fetal calf serum (FCS). The cells were incubated in a 5% CO_2_ atmosphere at 37 °C.

### Confocal microscopy experiments

KB cells were grown on Nunc™ Lab-Tek™ II Chamber Slide™ System (Thermo Scientific) at semi confluent concentration. One hour before treatment, EMEM culture medium was changed to RPMI medium without folic acid. Then, the cells were incubated for 4 h with 5 μM Dox or appropriate concentration of AuNPs to provide an equimolar concentration of Dox. After treatment, the cells were fixed in 1.5% formaldehyde and VectaShield DAPI stain containing anti-fade reagent was immediately added. Samples were refrigerated overnight and microscopic images were recorded. Confocal imaging was performed using a NIKON Eclipse Ti-1 microscope equipped in ×100 oil-immersed Plan Fluor objective (Nikon) and a Nikon A1 confocal system.

### Cytotoxicity analysis

An MTT test, which measures the activity of mitochondrial dehydrogenases and roughly corresponds to cellular viability, was used to evaluate the toxicity of the synthesized compounds. The assay was performed as described previously.^23^ In brief, KB cells were seeded a day before the experiment in 96-well microplates (TPP, Austria) at a density of 1 × 10^4^ cells per well in 100 μL of the EMEM culture medium. One hour before the treatment, the EMEM culture medium was changed to the RPMI medium without folic acid. Next, the cells were treated with AuNPs or Dox in the concentrations of 1 × 10^−6^ M and 5 × 10^−6^ M or vehicle (control) for 24 h or 48 h at 37 °C in a 5% CO_2_ humidified atmosphere. After incubation, 20 μL of MTT [3-(4,5-dimethyl-2-thiazolyl)-2,5-diphenyl-2*H*-tetrazoliumbromide] of 5 mg mL^−1^ stock solution in PBS (pH 7) was added to each well and incubated for 3 h at 37 °C. Then, the MTT solution was removed and remaining insoluble formazan crystals were dissolved in 100 μL DMSO. The absorbance was measured in the plate reader spectrophotometer (Infinite M200, Tecan, Austria) at a wavelength of 570 nm. The cell metabolic activity, which roughly relates to cell viability, was expressed as ratio of absorbance of the treated cells to the absorbance of the cells treated with vehicle, both after subtraction of the reagent control and multiplied by 100%. At least three independent experiments in six replicate wells were conducted.

### Statistical analysis

The differences between samples and the control were evaluated using GraphPad Prism 5.0 software (GraphPad Software Inc., USA). The toxicological data were evaluated by Kruskal–Wallis one way analysis of variance on ranks (ANOVA) followed by the *post hoc* Dunnet's method. The sensitivities of the cell lines were tested using a two-ways analysis of the variance followed by the post hoc Tukey's test. Differences were considered statistically significant when the *p*-value was <0.05.

## Results and discussion

### Characterization of the gold nanoparticles

**Fig. 1:**
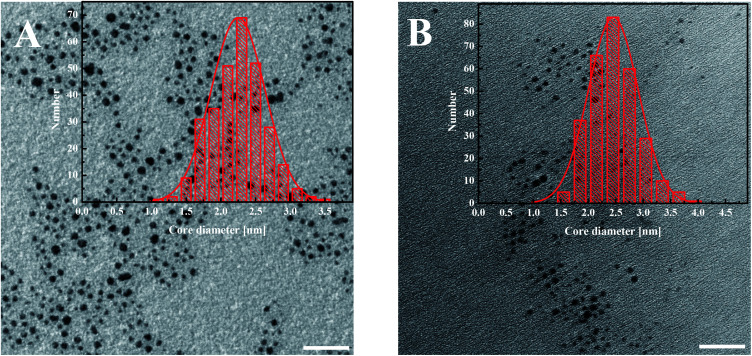
TEM images of (A) AuNPs-LA and (B) AuNPs-LA/LAFA. The scale bar is 20 nm. The histograms show the size distributions of the AuNPs evaluated from the TEM images.

The modified AuNPs were characterized by transmission electron microscopy (TEM), UV-Vis spectroscopy, thermogravimetric analysis (TGA) and voltammetry. The metallic core size of gold nanoparticles was evaluated from the transmission electron microscopy images. The average diameter of the gold cores obtained for AuNPs-LA is 2.2 ± 0.4 nm and for AuNPs-LA/LAFA is 2.5 ± 0.5 nm (Fig. 1).

**Fig. 2:**
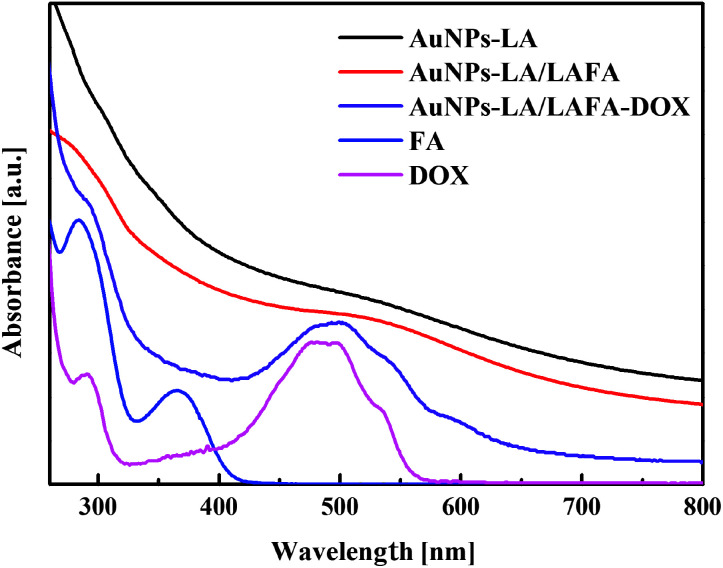
UV-Vis spectra of AuNPs-LA (black curve), AuNPs-LA/LAFA (red), AuNPs-LA/LAFA–Dox (violet), free FA (blue), free Dox (pink). The spectra are vertically shifted for better a visualization of the characteristic bands and regions of absorption.

The AuNPs-LA and AuNPs-LA/LAFA were characterized using optical and electrochemical methods. UV-Vis spectra of the obtained modified gold nanoparticles in solution show the continuous spectrum of gold nanoparticles in the visible region and signals corresponding to the free folic acid derivative and to the doxorubicin derivative (Fig. 2).

**Fig. 3:**
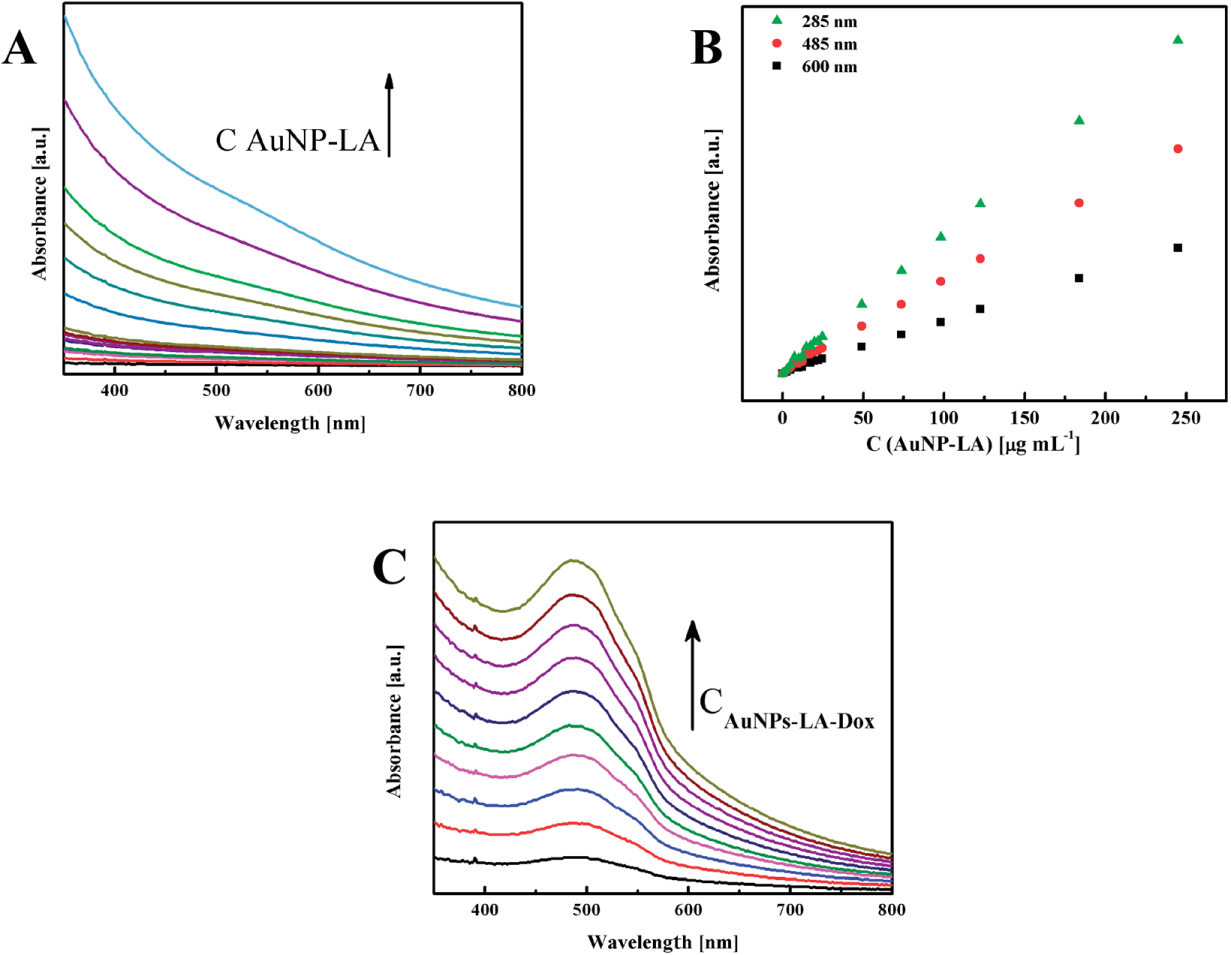
(A) UV-Vis spectra of AuNPs-LA with increasing concentrations and (B) calibration curves for various wavelengths. (C) Spectra for increasing concentrations of AuNPs-LA–Dox.

The evaluation of the AuNPs concentration and the concentration of the ligands on the AuNPs surfaces that is based on UV-Vis spectroscopy results (Fig. 3) is described in the ESI, S3.†

Thermogravimetric analysis confirmed the presence of LA and LAFA ligands on the nanoparticles surfaces (ESI, S4†). Additionally, on the basis of the subsequent inflection on the TGA curves, we can estimate the approximate content of ligands present on the AuNPs.

The presence of Dox on the LA and LA/LAFA modified AuNPs was confirmed using an electrochemical method since Dox is an electroactive molecule.^24^ The electrode processes of Dox are shown in Scheme S1-B (in ESI).†

The quinone moiety of Dox is reduced at negative potentials to hydroquinone, and electrochemical studies were then used for the detection of Dox in the coating of AuNPs, AuNPs-LA–Dox and AuNPs-LA/LAFA–Dox. Results were compared with the signals obtained for free Dox in a 0.15 M McIlvaine buffer solution, pH = 5.5. Gold nanoparticles coupled with Doxorubicin (*ca.* 1 μM) were added to the electrochemical cell and preconcentrated at the GC electrode surface by cycling the potential in the potential window of 0 to −0.7 V. Cyclic voltammograms (ESI, S5†) show the characteristic Dox reduction/oxidation peak couple at *ca.* −0.5 V *vs.* Ag/AgCl, which corresponds to the reduction of the quinone moiety to hydroquinone and its reoxidation in the reverse half-cycle. The Dox reduction peak in case of AuNPs-LA/LAFA–Dox appears at a slightly less negative potential than free Dox and AuNPs-LA–Dox due to the π–π interactions between the drug and FA groups. The reversible and stable pattern in the time signals of the Dox moiety attached to gold nanoparticles indicates an efficient coupling of Dox to the nanoparticles by the covalent bond. A non-decreasing doxorubicin activity was observed even after one month.

### Cell studies of the AuNPs based carriers

#### Fluorescence and confocal microscopy imaging of AuNPs treated KB (CCL-17) cells

**Fig. 4:**
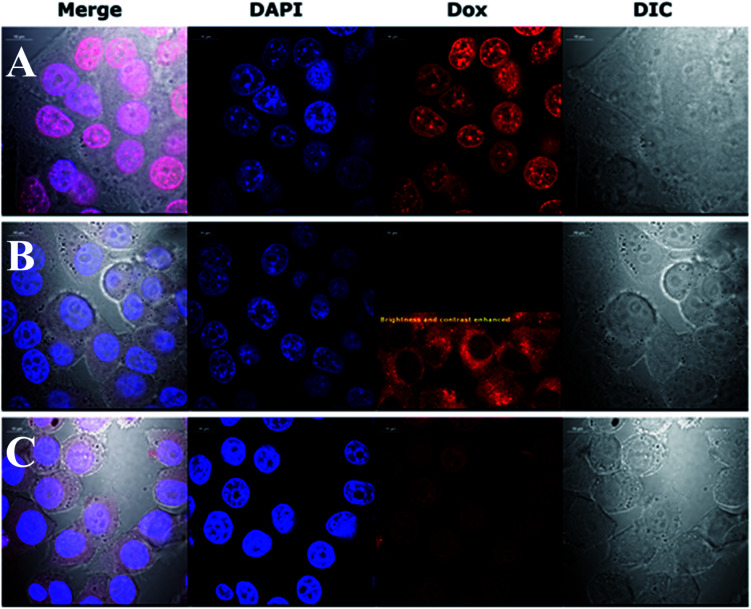
Confocal images showing how the presence of folic acid on an NP surface affects the internalization and nuclear accumulation of Dox. KB cells were fixed after a 4 h incubation with 5 μM Dox (1st row, A), AuNPs-LA–Dox (2nd row, B) and AuNPs-LA/LAFA–Dox (3rd row, C). The calculated Dox concentration in the AuNPs preparation equaled 5 μM. Cells nuclei of fixed cells were stained with DAPI dye (blue, 2nd column). Dox fluorescence (red, 3rd column). Cell bodies were imaged using transmitted light and Nomarski DIC contrast (gray, 4th column).

A continuous observation by fluorescence microscopy of live cells treated with AuNPs-LA–Dox for 22 h revealed that AuNPs-LA–Dox particles were hardly uptaken and accumulated in the cytoplasm (ESI, S6†). However, this results proved also the stability of amide bonding used to attach Dox to AuNPs-LA. Whereas, Dox alone freely migrated through the cytoplasm to the cell nucleus (Fig. 4, row A, 3rd column), the Dox fluorescence from AuNPs-LA–Dox was present predominantly in the cytoplasm (Fig. 4, row B, 3rd column) confirming that Dox was still attached to the AuNPs even 22 h after entering the cytoplasm (ESI, S6†).

This preliminary experiment showed that the most pronounced difference in intracellular Dox fluorescence of treated cells was observed 4–5 h after treatment (data not shown). Therefore, we decided to use a 4 h treatment time to perform high quality confocal images of fixed cells with stained nuclei. The results revealed large difference in the accumulation of Dox in the cytoplasm and nuclei (Fig. 4). Dox fluorescence (red) was the brightest in cells treated with Dox alone; then in AuNPs-LA/LAFA–Dox treated cells. Whereas, in AuNPs-LA–Dox treated cells it was only just visible. However, a duly analysis of red fluorescence on the AuNP-LA–Dox image indicated the presence of weak Dox signal in the cytoplasm, but not in the cell nucleus (Fig. 4, row B, 3rd column), that was in line with our observation using 22 h life cells imaging. No Dox fluorescence was seen in the control cells (not shown).

Confocal imaging revealed that folic acid enriched AuNPs were able to deliver Dox to the nucleus of the cell. Since we did not find Dox accumulation in cells treated with similar particles containing LA–Dox conjugate without folic acid addressing molecule, we suggest that the observed difference results from AuNPs-LA/LAFA–Dox binding to the folic acid receptor that enhances the transfer of those particles through the outer cell membrane and facilitates their incorporation into the nucleus. Boshnjaku *et al.* found that the folic acid receptor alpha (FRα) may be transferred through the nuclear envelope to reach DNA and act as a transcription factor. Additionally, an unbound free FRα was found in the nuclear envelope.^25^ Recently, Chaumet *et al.* showed that a substantial fraction of early endosomes may deliver particular plasma membrane receptors to the nucleus.^26^ Although Chaumet *et al.* did not study the FRα receptor, the proposed mechanism seems more universal and the direct delivery of FRα to the nucleus through the endosome-mediated transfer seems highly probable. Thus, it is plausible that the whole complex of AuNPs-LA/LAFA–Dox, once bound to the plasma membrane FRα receptor, is trapped inside the endosome, which is in turn transported to nucleus. This would explain why we have not seen the nuclear accumulation of AuNPs-LA–Dox even after 22 h of incubation. The AuNPs-LA–Dox nanoparticles were possibly uptaken by a non-specific mechanism that resulted in a much slower AuNPs accumulation in the cytoplasm and apparent lack of cytotoxicity. The proposed above folate receptor dependent mechanism of nucleus transport prevails in case of AuNPs-LA/LAFA–Dox bioconjugates and results in better nucleus penetration and enhanced toxicity (see below).

### Cytotoxicity

**Fig. 5:**
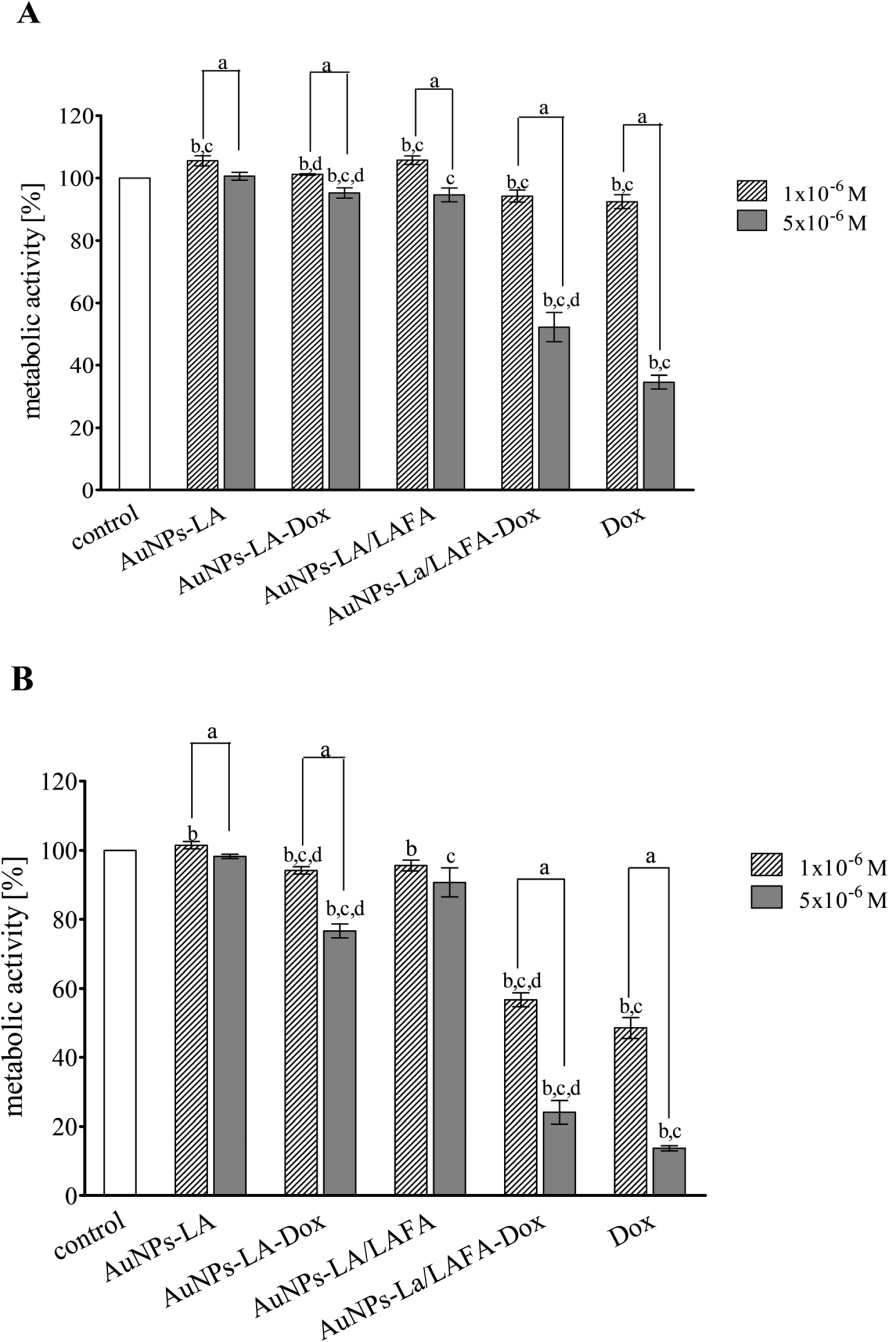
Toxicity of the AuNPs conjugates and free doxorubicin to KB (CCL-17) cells treated for 24 h (A) and 48 h (B). Data are expressed as a percent of the control and the mean ± SD from three independent experiments. *P* < 0.05 (‘a’ denotes a statistically significant difference between the 1 × 10^−6^ M and 5 × 10^−6^ M concentrations; ‘b’ denotes a statistically significant difference between the 24 h and 48 h treatment, ‘c’ denotes a statistically significant difference *versus* the control group; and ‘d’ denotes a statistically significant difference between the AuNPs–Dox complexes and Dox alone).

The cytotoxic activity of tested compounds was investigated on KB cells using the MTT assay (Fig. 5). AuNPs-LA and AuNPs-LA/LAFA were not cytotoxic in the concentrations used. AuNPs-LA–Dox complex expressed small, but statistically significant toxicity in concentration 5 × 10^−6^ M after 48 h incubation. This concentration reduced metabolic activity of KB cells to 76% of control value. On the contrary, treatment of the cells with AuNPs-LA/LAFA–Dox and Dox alone resulted in a marked reduction of metabolic activity in a dose- and time-dependent manner. The highest used concentration after 48 h incubation reduced the metabolic activity of KB cells to 25 and 13% for AuNPs-LA/LAFA–Dox and Dox, respectively. However, the further studies of toxicity are needed to obtain additional information on mutagenicity or genotoxicity of the developed drug carriers in view of several literature reports.^27–29^

## Conclusions

We described a successful procedure for the preparation of bifunctional gold nanoparticles for drug delivery. The preparation of a lipoic acid derivative of folic acid soluble in a synthetic medium allowed the attachment of the target molecule to the AuNPs and increased the carrier solubility in aqueous solutions. The carrier was made more hydrophilic by the subsequent step of self-assembly of free lipoic acids in the voids not covered by the folic derivative. To the modified nanoparticles, the cytotoxic drug Dox could be bound *via* covalent (amide) bond. The replacement of the common ligand exchange reaction with the *in situ* formation of thiol modified AuNPs improves the control of the number of sites on the AuNP occupied by the drug. More importantly, the amide bonding slowed down the release of the drug and increased the carrier stability in the cell environment.

Electrochemical and UV-Vis spectroscopy experiments proved the presence of doxorubicin on the surface of AuNPs and enabled the estimation of the gold nanoparticles concentration and surface coverage by FA and LA derivatives.

The linker used in the present work did not disturb the folic acid binding to its receptor on the membranes of the KB cells. The synthesized AuNPs coupled with Dox easily penetrated to the cell nucleus and expressed toxicity comparable with that of Dox alone. Whereas, the same AuNPs without addressing unit (FA) did not show any significant internalization or cytotoxicity. All together it indicates that folic acid derivative was necessary for the proper up-take of the synthesized nanoparticles by the cells while the amide bonding provided sustained release of the drug limiting it mainly to the nucleus. Results showed also that AuNPs with a protective layer made of lipoic acid and/or lipoic acid derivative of an “addressing molecule” are transferred through the nuclear envelope to reach DNA, likely *via* receptor mediated mechanism.

The application of these targeted ultrasmall nanoparticles opens a new route for the delivery of toxic drugs. A tight covalent amide bond used to attach Dox to LA moiety resulted in a stable bioconjugate that persist at least 22 h in the cytoplasm. Thus, it is anticipated that once cell dies, the drug is released in unchanged form, ready to interact with another cell. This reciprocal mechanism of action should increase the drug efficacy and limit its cytotoxicity to target receptor overexpressing cells rather than all cells present in vicinity, as in case of free Dox released from non-covalent bound bioconjugates. In addition, Dox tightly attached to AuNPs is expected to has less systemic toxicity than its free form, readily penetrating all organs of the body. Finally AuNPs with attached Dox are expected to have slower body clearance than Dox alone, thus should be more efficient in the cancer treatment. *In vitro* studies on cancer cells culture proved that such nanoparticles are promising drug carriers and they are worth to be further investigated in *in vivo* experiments.

## Conflicts of interest

There are no conflicts to declare.

## Supplementary Material

RA-008-C8RA01107A-s001
