# Supplementary material for: Towards potent but less toxic nanopharmaceuticals – lipoic acid bioconjugates of ultrasmall gold nanoparticles with an anticancer drug and addressing unit
Source: RSC Adv. 2018 Apr 19;8(27):14947–57. doi: 10.1039/c8ra01107a (PMC9079921; doi:10.1039/c8ra01107a)
Supplement: RA-008-C8RA01107A-s001 [file RA-008-C8RA01107A-s001.pdf]

Electronic Supplementary Material (ESI) for RSC Advances.
This journal is © The Royal Society of Chemistry 2018

Supplementary Information

Towards potent but less toxic nanopharmaceuticals – Lipoic acid bioconjugate of ultrasmall gold nanoparticles with anticancer drug and addressing unit

*Maciej Dzwonek[a], Dominika Załubiniak[a], Piotr Piątek[a], Grzegorz Cichowicz[b], Sylwia Męczynska-Wielgosz[c], Tomasz Stępkowski[c], Marcin Kruszewski[c,d], Agnieszka Więckowska[a], Renata Bilewicz[a]**

[a] Faculty of Chemistry, University of Warsaw, Pasteura 1, 02-093, Warsaw, Poland

[b] Czochralski Laboratory of Advanced Crystal Engineering, Biological and Chemical Research Centre, Faculty of Chemistry, University of Warsaw, Żwirki i Wigury 101, 02-089 Warsaw, Poland

[c] Center for Radiobiology and Biological Dosimetry, Institute of Nuclear Chemistry and Technology, Dorodna 16, 03-195 Warszawa, Poland

[d] Department of Molecular Biology and Translational Research, Institute of Rural Health, Jaczewskiego 2, 20-090 Lublin, Poland

*Corresponding author: Renata Bilewicz
E-mail address: bilewicz@chem.uw.edu.pl
Faculty of Chemistry, University of Warsaw, Pasteura 1, 02-093, Warsaw, Poland
Tel: +48 22 55 26 357

S1. Schemes of doxorubicin formula and doxorubicin red-ox reactions

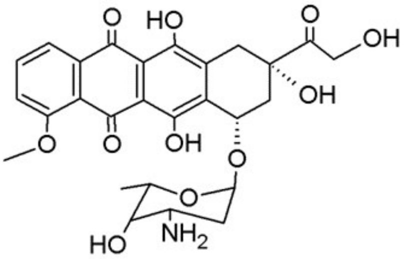

Scheme S1 - A. Doxorubicin (Dox) structure.

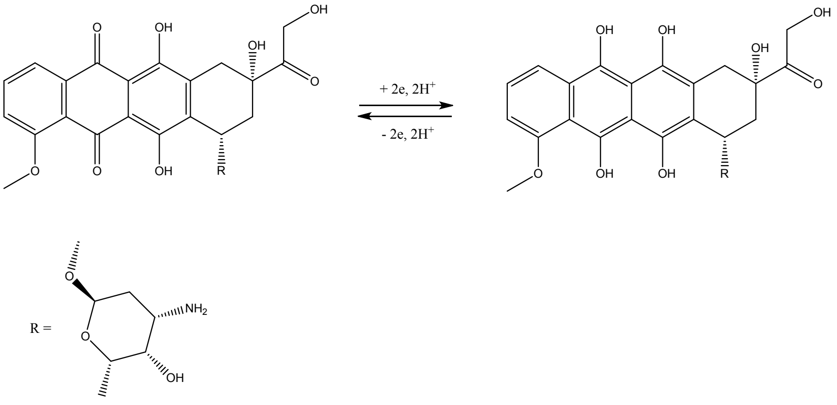

Scheme S1 - B: Electrochemical reactions of Dox.

S2. **NMR spectra of the transition and final products of LA-FA synthesis**

Protection of ethylenediamine using t-butyloxycarbonyl anhydride

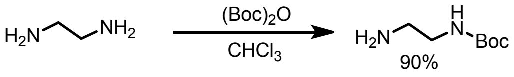

^{1}H NMR (300 MHz, $CDCl_3$) δ: 4.94 (s, 1H), 3.17-3.11 (q, 2H), 2.79-2.72 (t, 2H), 1.42 (s, 9H), 1.16 (s, 2 H).

Modification of folic acid by protected ethylenediamine.

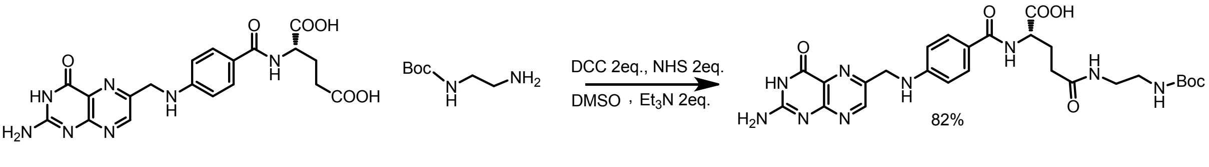

^{1}H NMR (300 MHz, DMSO) δ: 11.46 (s, 1H), 8.64 (s, 1H), 8.20-7.86 (m, 2H), 7.68-7.62 (m, 2H), 7.30-6.72 (m, 3H), 6.66-6.63 (d, 2H), 4.48 (s, 2H), 4.27 (m, 1H), 3.09-2.85 (m, 4H), 2.28-1.85 (m, 4H), 1.35(s, 9H).

^{13}C NMR (300 MHz, DMSO) δ: 172.72, 166.68, 161.64, 156.08, 154.18, 151.23, 151.09, 148.57, 129.66, 129.32, 128.31, 121.59, 111.87, 78.27, 52.84, 46.43, 46.02, 40.94, 39.27, 32.88, 31.51, 28.68, 9.86.

MS (E $^{-}$): 582.40

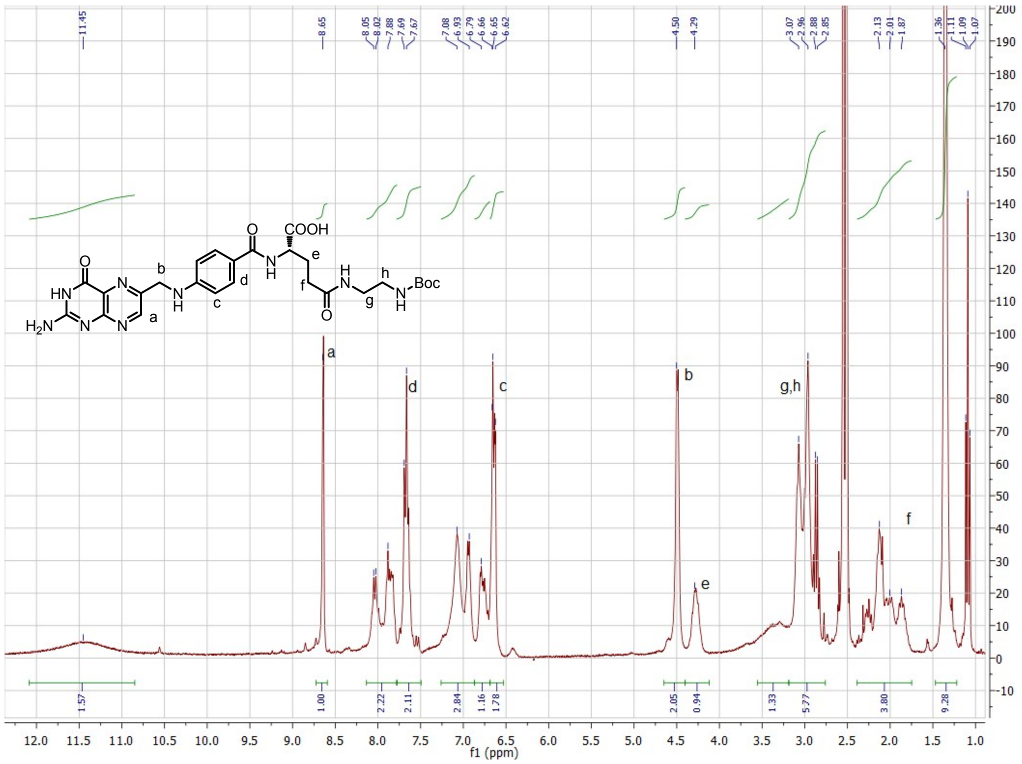

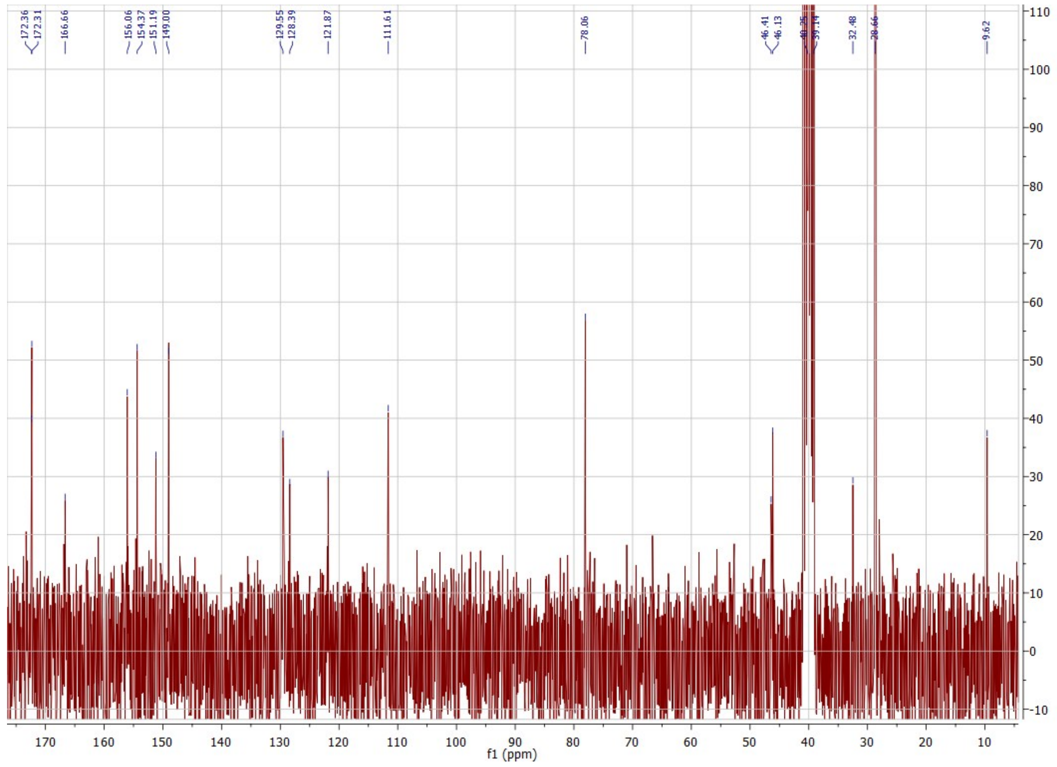

Activation of lipoic acid by N-disuccinimydylcarbonate

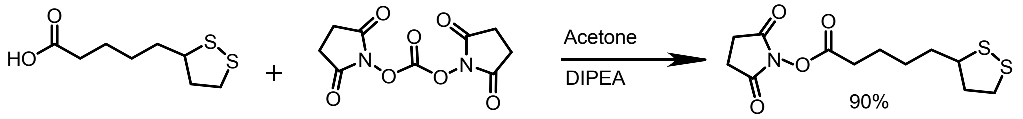

^{1}H NMR (300 MHz, $CDCl_3$) δ: 3.62-3.53 (m, 1H), 3.19-3.09 (m, 2H), 2.83 (s, 4H), 2.66-2.60 (m, 2H), 2.5-2.43 (m, 1H), 1.96-1.89 (m, 1H), 1.81-1.54 (m, 6H)

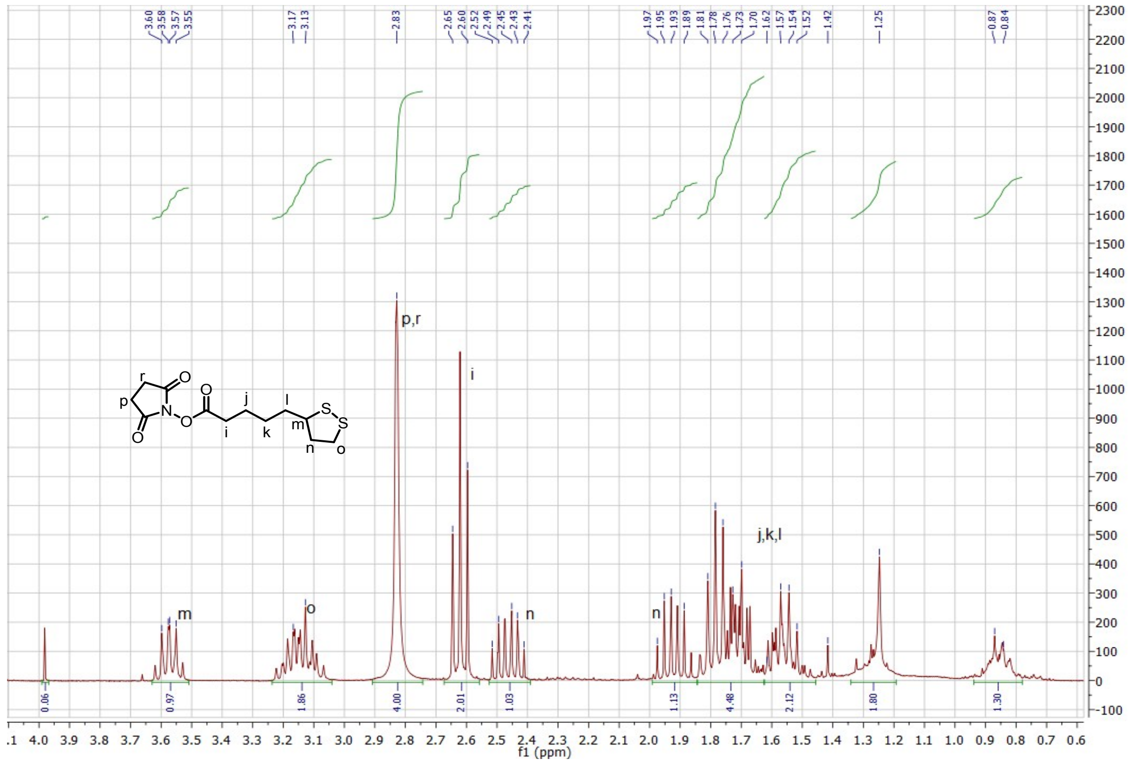

Conjugation of lipoic and folic acid derivative. LA-FA

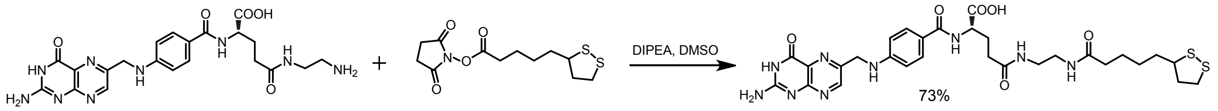

^{1}H NMR (300 MHz, DMSO) δ: 8.63 (s, 1H), 8.14-7.88 (m, 2H), 7.68-7.62 (m, 2H), 7.17 (bs, 2H), 6,91 (bs, 2H), 6.65-6.63 (m, 2H), 4.48 (bs, 2H), 4.26 (bs, 1H), 3.59-3.49 (m, 1H), 3.41-3.34 (m, 1H) 3.36-2.92 (m, 6H), 2.89-2.82 (m, 2H), 2.26-1.80 (m, 8H), 1.69-1.39 (m, 6H), 1.36-1.23 (m, 3H)

^{13}C NMR (300 MHz, DMSO) δ: 172.53, 167.10, 161.88, 160.00, 156.89, 154.66, 151.30, 148.96, 129.55, 129.19, 128.42, 121.83, 111.73, 65.38, 56.57, 46.39, 45.99, 35.71, 34.56, 28.79, 25.42, 19.20, 15.63, 9.97

MS (ES$^-$): 670.22

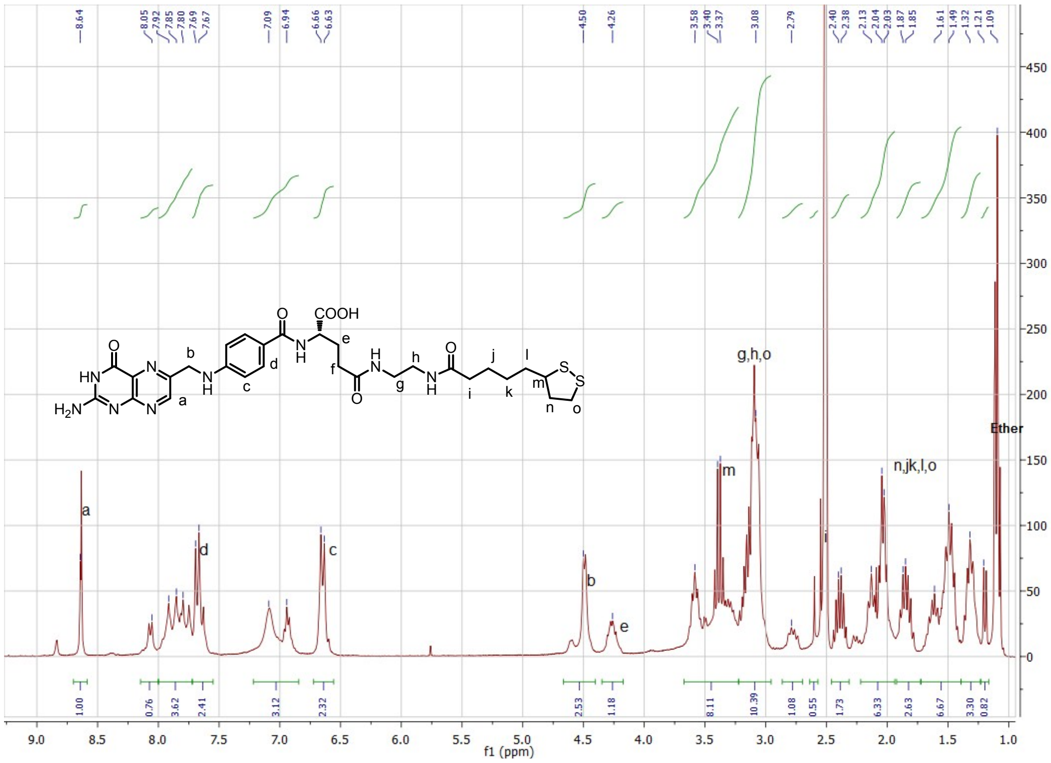
g,h,o
m
n,jk,l,o
Ether
a
d
c
b
e
f1 (ppm)

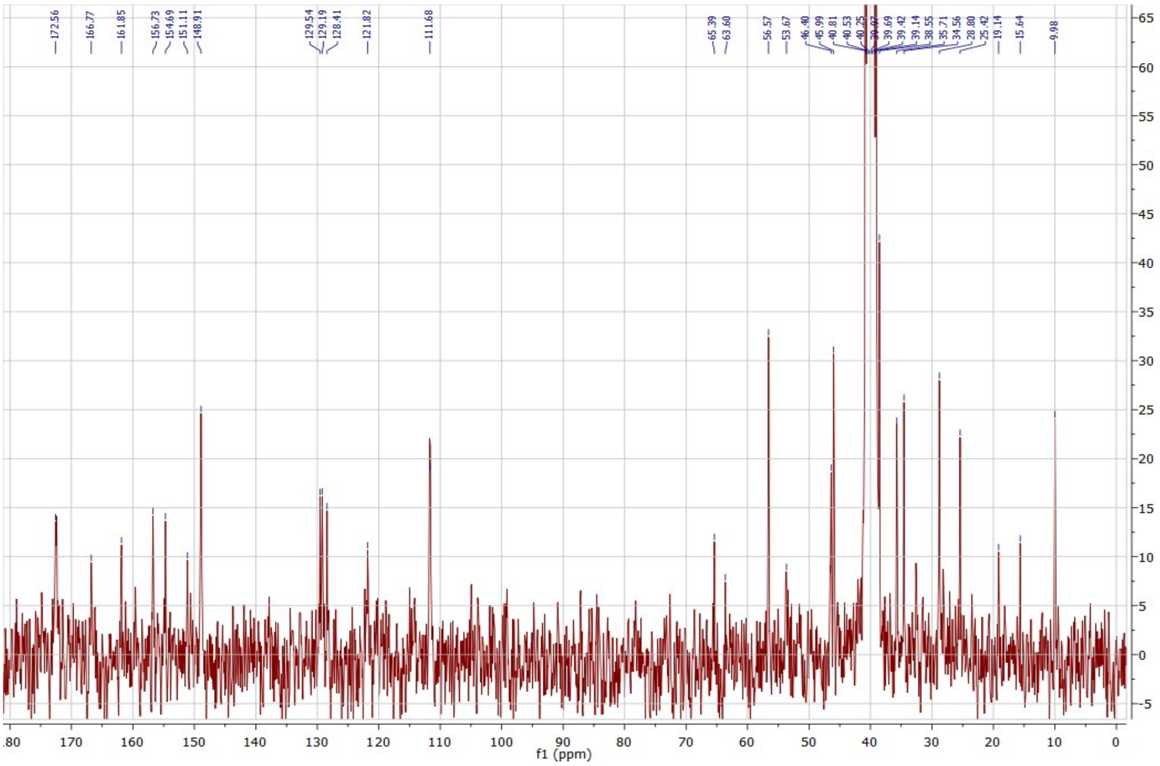
f1 (ppm)

S3. Evaluation of AuNPs concentration in the solution

Generally AuNPs absorb light in the whole UV-Vis region and the analysis is even harder when multiply chromophore ligands are attached to the nanoparticles' surface. On the other hand the dried samples often have a tendency to aggregate upon drying procedure. Here the method of estimation A series of solutions of gold nanoparticles was prepared, assuming that the whole amount of gold precursor used in synthetic procedure is converted to gold nanoparticles. The calibration curves were prepared using three wavelength values, which were chosen based of UV-Vis spectra of AuNPs-LA, free folic acid and free doxorubicin. Due to the continuous absorption of AuNPs in the whole UV-Vis range, for AuNPs-LA we choose a region where neither folic acid or doxorubicin do not show any absorption signals. For folic acid a wave of 285 nm was selected, for doxorubicin a 485 nm and for AuNPs-LA-600 nm (Fig. 3). It allows to estimate concentration of gold nanoparticles (AuNPs-LA/LAFA) and ligands (FA) separately as a subtraction of gold nanoparticles background. After the doxorubicin attachment analogous method was used to estimate doxorubicin content on AuNPs-LA/LAFA-Dox. For example, using absorbance value of nanoparticles sample at 600 nm (either AuNPs-LA or AuNPs-LA/LAFA), there is a possibility to estimate concentration of nanoparticles sample only, and then using calibration curve for AuNPs-LA at 285 nm, calculation of approximate contribution of nanoparticles in superimposed signal in AuNPs-LA/LAFA.

$$A_{285nm\ FA} = A_{285\ AuNPs-LA/LAFA} - A^{*}_{285\ nm\ AuNPs-LA}$$

$A_{285\ nm\ FA}$ – estimated absorbance of folic acid ligand on nanoparticles,

$A_{285\ nm\ AuNPs\text{-}LA/LAFA}$ – absorbance of AuNPs–LA/LAFA sample,

$A^{*}_{285\ nm\ AuNPs\text{-}LA}$ – calculated absorbance of AuNPs-LA from calibration curves.

A

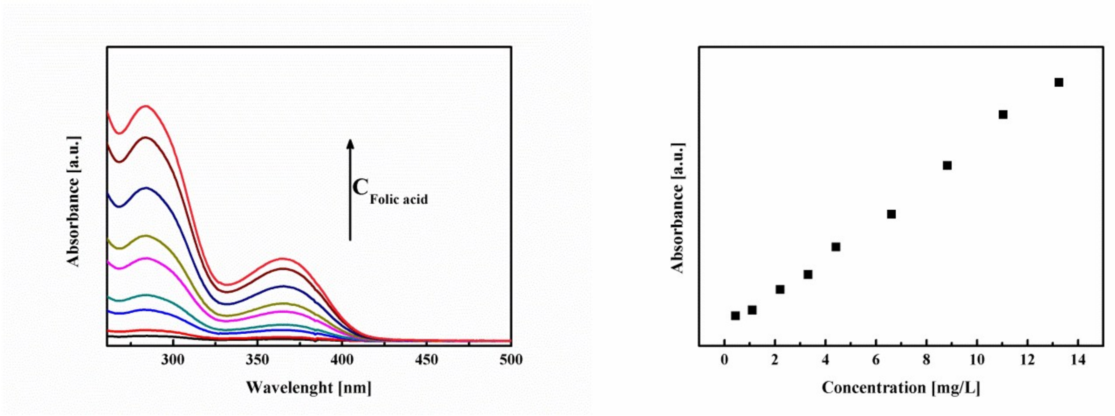

B

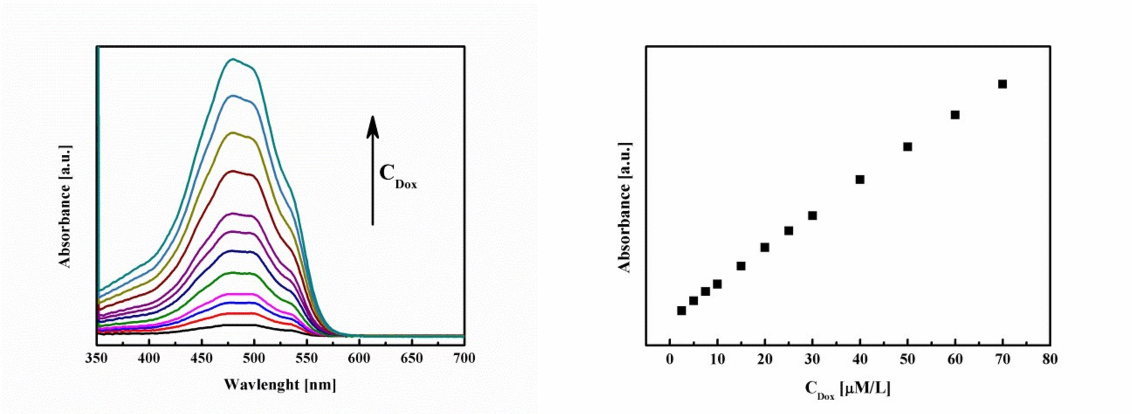

Fig. S3 UV-Vis spectra of A) folic acid (in 0.05 M NaOH) and B) doxorubicin (in DI water) at different concentrations used for the calibration curves.

S4. TGA analysis

TG and DTG (1st derivative) curves. for AuNPs-LA and AuNPs-LA/LAFA as well as curves for pure ligands (LA and LAFA) are shown in Figure S4. Decomposition of AuNPs-LA (Figure S4 A) starts in 167°C, with one strong, characteristic signal on DTG curve also observed on DTG curve for LA ligand (Figure S4 B). The maximum rate of the process is observed at 215°C. The decomposition of AuNPs-LA/LAFA is more complex, and proceeds in three decomposition steps: (1) LA ligand, (2) amide bonds between LA and LAFA moiety and (3) the rest of organic compounds (Figure S4 C). All of these three signals can be distinguished in LA-LAFA TG/DTG curve (Figure S4 D). The weight loss for this sample starts in 177°C, whereas second step occurs in 256°C and third in 394°C.

In both DTG curves of AuNP samples, in the temperature range above 500°C two peaks were seen corresponding to the decomposition of two S-Au bonds. For the AuNPs-LA sample, TG data showed 5.91% weight loss at 564°C while weight loss for the AuNPs-LA/LAFA sample was higher, 16.97% and started in 480°C. However, for the second AuNPs sample decomposition of two S-Au bonds signal can overlap with the signal of decomposition of the rest of organic compounds. Therefore, the decomposition of two S-Au bonds can start at higher temperature, as in AuNPs-LA curve in 564°C. Basing on this assumption the mass ratio of LA or FA to Au was calculated. The weight loss for this temperature was 12.42 %. Using value above, the evaluated LA ligand to Au mass percent ratio was 69.8% and FA to Au mass percent ratio was 4.9%. For the AuNPs-LA obtained LA to Au ratio was 23.9%.

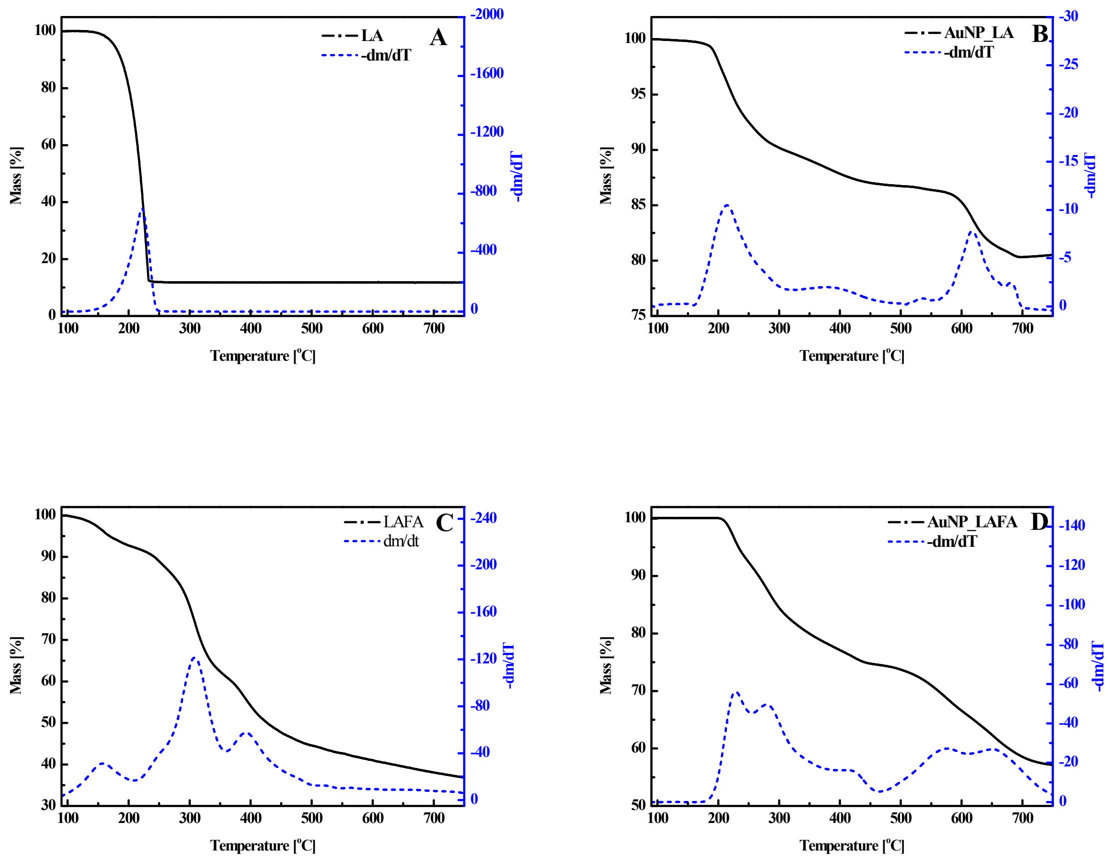

Fig. S4: TG (black solid) and DTG (1st derivative, blue dotted) curves for (B) AuNPs-LA and (D) AuNPs-LA/LAFA in comparison with curves for pure ligands: (A) LA and (C) LAFA.

S5. Voltammetry detection of Dox presence on the AuNPs surfaces

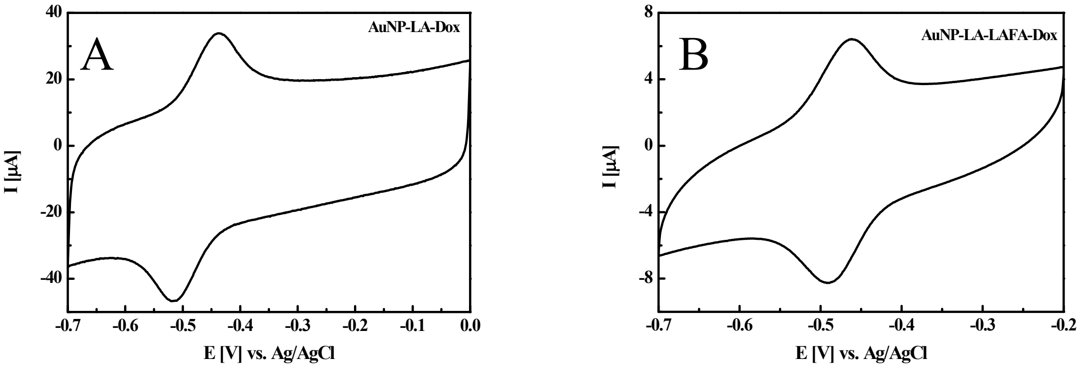

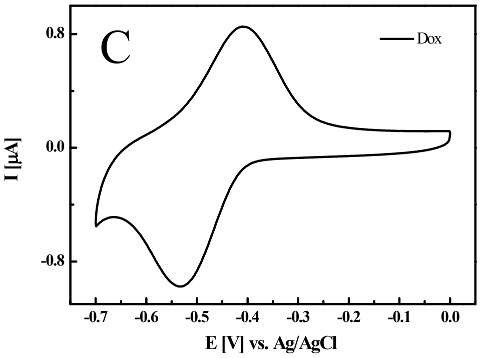

Fig. S5. Cyclic voltammograms in 0.15 M McIlvaine buffer solution, pH = 5.5 for: A) AuNPs-LA-Dox, B) AuNPs-LA/LAFA-Dox, C) free Dox. Scan rate: 20 mVs^{-1}.

S6. Fluorescence microscopy of live cells treated with AuNPs-LA-DOX for 22 h.

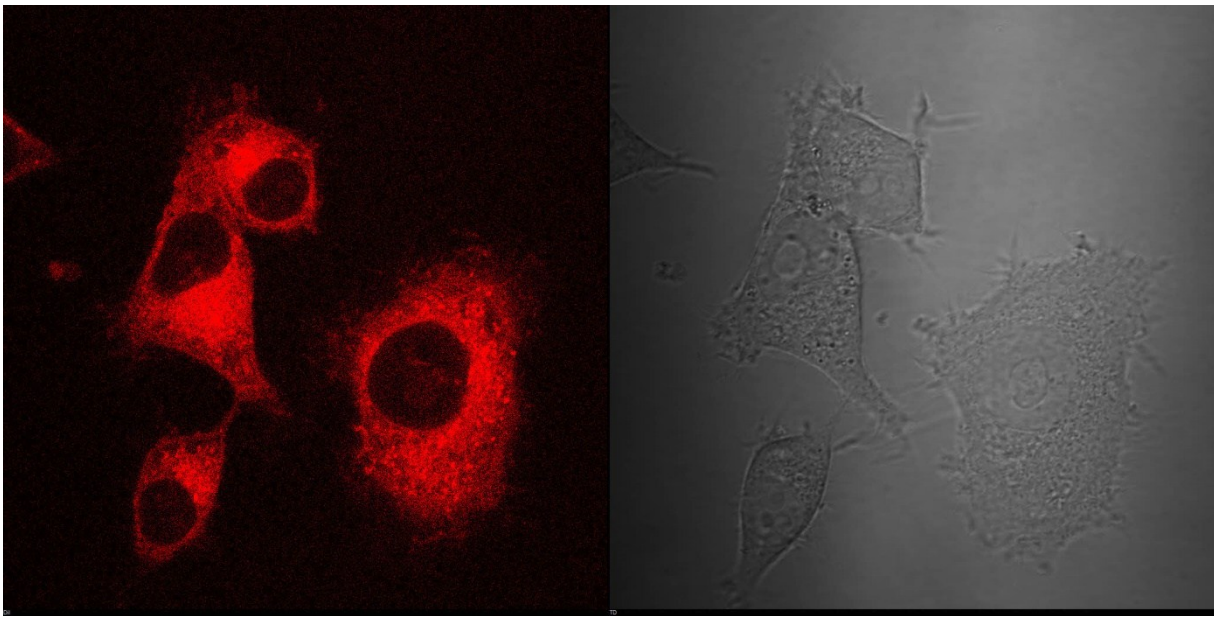

Fig. S6. Doxorubicin fluorescence in KB cells treated with AuNPs-LA-Dox for 22 h.
